# Reusable snorkel masks adapted as particulate respirators

**DOI:** 10.1371/journal.pone.0249201

**Published:** 2021-04-05

**Authors:** Henry Seligman, Sameer Zaman, David S. Pitcher, Matthew J. Shun-Shin, Freya Hepworth Lloyd, Vitaliy Androschuk, Sayan Sen, Rasha Al-Lamee, David M. Miller, Harry W. Barnett, Gulam S. Haji, Luke S. Howard, Sukhjinder Nijjer, Jamil Mayet, Darrel P. Francis, Oscar Ces, Nicholas W. F. Linton, Nicholas S. Peters, Ricardo Petraco

**Affiliations:** 1 National Heart and Lung Institute, Imperial College London, London, United Kingdom; 2 Department of Cardiology, Imperial College Healthcare NHS Trust, London, United Kingdom; 3 Imperial Centre for Cardiac Engineering, Imperial College London, London, United Kingdom; 4 Advanced Hackspace, Imperial College London, London, United Kingdom; 5 Department of Chemistry, Imperial College London, London, United Kingdom; Hong Kong Polytechnic University, HONG KONG

## Abstract

**Introduction:**

During viral pandemics, filtering facepiece (FFP) masks together with eye protection form the essential components of personal protective equipment (PPE) for healthcare workers. There remain concerns regarding insufficient global supply and imperfect protection offered by currently available PPE strategies. A range of full-face snorkel masks were adapted to accept high grade medical respiratory filters using bespoke-designed 3D-printed connectors. We compared the protection offered by the snorkel to that of standard PPE using a placebo-controlled respirator filtering test as well as a fluorescent droplet deposition experiment. Out of the 56 subjects tested, 42 (75%) passed filtering testing with the snorkel mask compared to 31 (55%) with a FFP3 respirator mask (p = 0.003). Amongst the 43 subjects who were not excluded following a placebo control, 85% passed filtering testing with the snorkel versus to 68% with a FFP3 mask (p = 0.008). Following front and lateral spray of fluorescence liquid particles, the snorkel mask also provided superior protection against droplet deposition within the subject’s face, when compared to a standard PPE combination of FFP3 masks and eye protection (3.19x10^8^ versus 6.81x10^8^ fluorescence units, p<0.001). The 3D printable adaptors are available for free download online at https://www.ImperialHackspace.com/COVID-19-Snorkel-Respirator-Project/.

**Conclusion:**

Full-face snorkel masks adapted as particulate respirators performed better than a standard PPE combination of FFP3 mask and eye protection against aerosol inhalation and droplet deposition. This adaptation is therefore a promising PPE solution for healthcare workers during highly contagious viral outbreaks.

## Introduction

Following the global SARS CoV-2 coronavirus (COVID-19) outbreak in March 2020, the World Health Organisation (WHO) [1] and the Center for Disease Control and Prevention (CDC) [2] have published guidelines recommending the use of extensive personal protective equipment (PPE) by healthcare workers. The magnitude and timescale of the COVID-19 outbreak has created an unprecedented global demand for PPE, in particular for filtering facepiece (FFP) masks. Together with eye protection provided by visors or glasses, FFP masks are recommended for impeding viral transmission via small respiratory droplets (aqueous particle greater than 5–10μm) and aerosols (liquid or solid particles suspended in air <5μm) [1–6]. However, global supply of PPE equipment has not met the demand [7, 8] and there remain uncertainties as to whether the currently recommended PPE strategies provide adequate protection to all staff [9, 10].

In this study, we investigate whether full-face snorkel masks adapted with high grade respiratory filters via 3D-printed connectors can provide equivalent protection to currently recommended PPE with respect to particulate filtration and droplet deposition. Potential benefits of such adaptation are the universal availability and cost-effectiveness of reusable snorkels, filters and 3D-printed adaptors.

## Methods

This study was performed during routine mask testing during the peak of the COVID pandemic and is a quality improvement project. It accordingly did not require specific ethical approval by the local review board. All participants provided informed verbal consent to take part. It was reviewed and approved as a quality improvement project by the Hospital Audit Committee.

### Snorkel mask, adaptors and filters

Full technical details of the parts used, design and 3D printing methods of adaptors, as well as the experiments undertaken can be found in the S1 Appendix. Two different brands of commercially available adult full-face snorkel diving masks with two different types of outflow shape were used. One elliptical (Mosfiata, Shenzhen China) and one round (X99, purchased through Amazon.co.uk). Two different types of air filters (Filta-Therm HME filter, Intersurgical, Wokingham, UK and 3M P3R 6035 filter, Saint Paul, Minnesota, USA) were used. Both are P3 graded with filtration rates above 99.9% for viruses and bacteria [11, 12] and were chosen because of their universal availability and brand reputation. To each snorkel mask, a pair of filters were connected via adapters which were designed and 3D printed in house, at the Imperial College Advanced Hackspace. The 3D printable devices are available for free download at https://www.ImperialHackspace.com/COVID-19-Snorkel-Respirator-Project/. The FFP masks used were disposable FFP3 respirators (1873V, 3M, Saint Paul, Minnesota, USA and Medline NR-EN1 respirator mask, Medline, Northfield, Illinois).

### Filtering test experiment

We designed an experiment to test the performance of adapted snorkel mask respirators against FFP3 masks under qualitative testing conditions most commonly used on the front line during the COVID-19 pandemic. Subjects were recruited from the cardiology and radiology departments at a large teaching hospital who were referred for routine mask fit testing. The sole inclusion criterion was being a member of staff at the trust. Exclusion criteria were being unwilling or unable to wear the PPE or to complete the testing protocol. This sample could be considered representative of the larger population of healthcare workers and a reasonable range of ages, genders and disciplines were covered (Table 1) however those with certain disabilities or face shapes might not be able to use the mask or to complete the testing protocol. Each participant was informed that they would be taking part in a standard mask fit test as required by the trust but asked if they would be willing to undergo extra steps to help us understand the performance of the new masks and the test itself. The steps were outlined verbally. It was made clear that if they did not want to take part a routine test would be offered freely. They were asked to complete and sign a departmental audit form however this was not retained by the study team. Verbal consent was documented on the anonymised data spreadsheet by the investigator. No participants were unwilling to consent however one withdrew consent after experiencing claustrophobia and was withdrawn from study and the analysis.

**Table 1:** Participant and equipment characteristics for the filtering test. Here are summarised the baseline characteristics of the participants, their professional roles and the types of masks used in the filtration experiment.

Participant characteristics	Number (%)
**Included in analysis**	**56**
Previously passed FFP3 fit test	33 (59)
Average Age (years)	36.8
Male Sex	25 (45)
Sensitive to Denatonium	46 (82)
Sensitive to Saccharine	10 (18)
Participants wearing glasses	8 (14)
Participants with beard	3 (5)
**Participant Role**	
Doctor	19 (34)
Radiographer	16 (29)
Nurse	8 (14)
Porter	3 (5)
Healthcare assistant	3 (5)
Sonographer	2 (4)
Modality assistant	1 (2)
Technologist	1 (2)
Physiologist	1 (2)
Medical student	1 (2)
Nurse endoscopist	1 (2)
**Equipment used for filtering test**	
**Snorkel mask**	
Mosfiata (elliptical connector)	39/56 (70)
X99 (round connector)	17/56 (30)
**FFP3 mask**	
Foldable 3M 1873V mask	56/56 (100)

Abbreviations: FFP, Filtering face piece

The filtration capacity of the adapted snorkel mask was compared to that of a standard FFP3 mask using a modified version of the standard qualitative respiratory protective equipment (RPE) testing protocol which is routinely used to test the fitting of FFP masks in healthcare workers at our trust (Imperial College NHS Trust) [13]. Full details of the method can be found in the S1 Appendix. In summary, each participant was randomly allocated to sequential testing with both snorkel mask and standard FFP3 mask. Following the introduction of aerosolised solutions (bitter denatonium *or* sweet saccharine) and placebo (water) into a hood, participants were asked whether they could taste it, whilst performing a standardised protocol of actions (normal breathing, deep breathing, head movement, speaking and bending). Those reporting to taste the solution at any stage, were considered to have failed the test. Those who tasted the placebo aerosol (false failures) were excluded in a pre-specified analysis.

### Droplet deposition experiment

In order to compare the snorkel mask’s ability to provide a physical barrier against droplet-based viral transmission with that of a standard FFP3 mask, fluorescent depositions of droplets observed under ultraviolet (UV) light were used to visually compare the protection offered by the adapted snorkel mask to that achieved by guideline-recommended [1, 3] standard PPE. Florescent powder (Golden Yellow Fluorescent Powder, Flint Hire & Supply Ltd., London, UK) was suspended in 30ml of low viscosity oil (Johnson’s baby oil, Johnson & Johnson GmbH, Neuss, Germany) and placed in a handheld triggered spray bottle. The participant was one of the investigators and has given written informed consent (as outlined in the PLOS consent form) to publish these case details. The participant was sprayed by a clinical researcher at a distance of 50cm, in each of four positions: front, right side, left side and back of head, whilst wearing the adapted snorkel mask and different types of PPE combinations–all in accordance with national and international guidelines [1–3]. The participant was photographed before and after fluorescence spraying as well as after appropriate PPE removal, using a Canon EOS 700D DSLR camera and Canon EF-S 18-55mm lens (Canon Inc., Tokyo, Japan). For the pictures under UV light, a battery-powered 68 LED bulb, 395nm handheld UV flashlight was used (Lighting Ever Ltd., Birmingham, UK). Full details of the experiment are presented in the S1 Appendix.

The magnitude of face fluorescence deposition after PPE doffing was quantified with a biological-image analysis software (ImageJ on the Fiji distribution) [14] using a protocol adapted by Howard et al [15] (see S1 Appendix for detailed methodology).

### Data analysis

Results of filtering test pass rates are expressed as proportions and percentages. The McNemar test was used to assess the differences in proportions of pass or fail rates between the two masks. Fluorescence data was presented as mean corrected total fluorescence units. An independent samples t-test was used for comparison.

## Results

### Filtering capacity testing

Between 12^th^ and 28^th^ May 2020, 57 healthcare workers were fit tested. Their baseline characteristics are summarised in Table 1. One participant was excluded from the analysis as they became claustrophobic shortly after test commenced.

**Fig 1:**
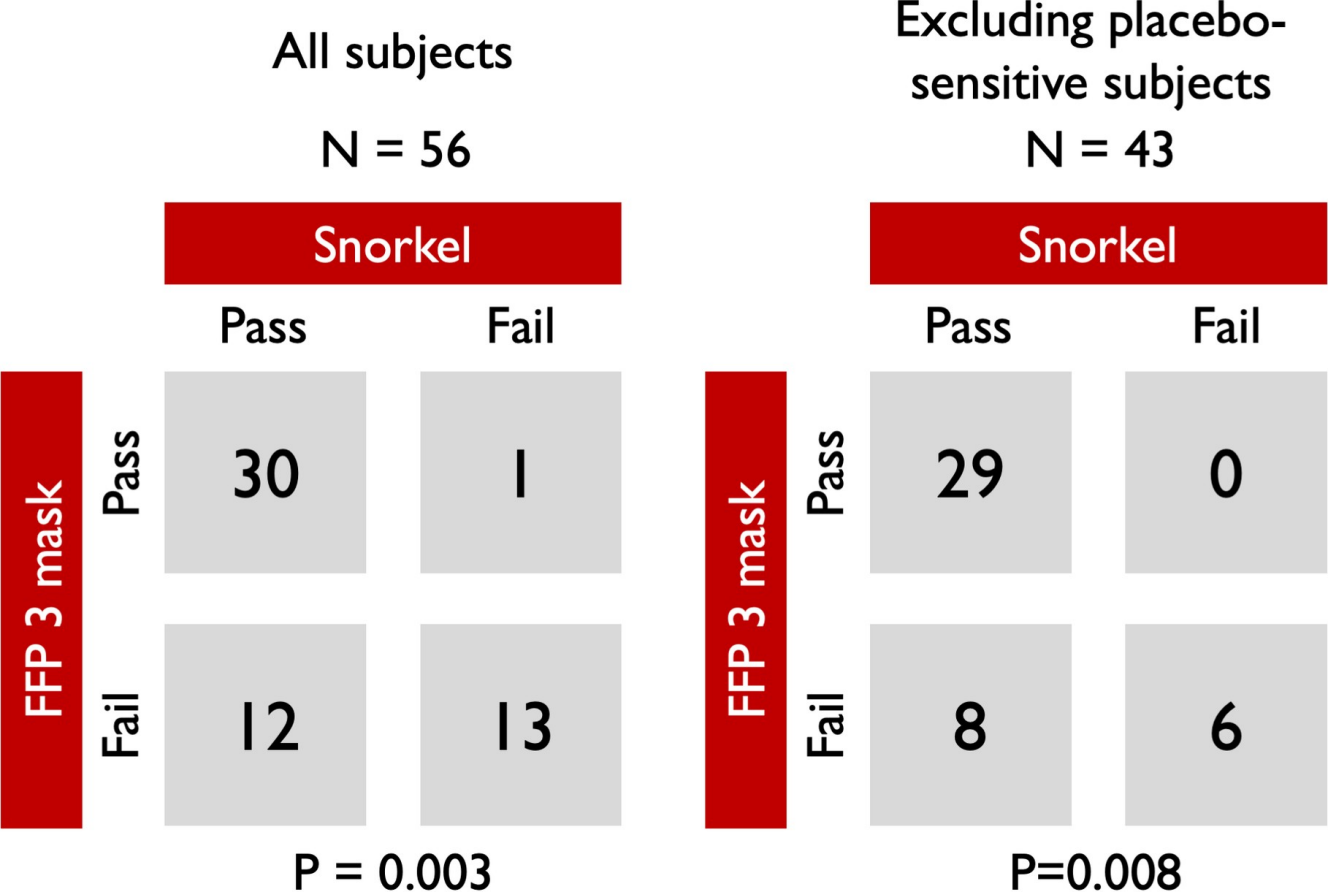
Comparison of fit testing pass rates between the adapted snorkel mask and standard FFP3 mask. Left panel: all participants. Right panel: excluding those sensitive to placebo.

Across all participants, 42/56 (75%) passed filtering testing with the snorkel mask compared to 31/56 (55%) with standard FFP3 respirator mask. 30 out of 56 (54%) passed the test for both masks, whilst 13/56 (23%) failed both. Of the 13 participants (23%) who only passed with one mask, significantly more passed with the snorkel mask (12/13 vs 1/13, p = 0.003) (Fig 1). When participants who failed the placebo test (those who subjectively declared tasting a water aerosol, n = 13) were excluded from the analysis, the improved performance of the snorkel mask became more evident. Amongst those who passed the FFP3 mask (n = 29), no failure was observed with the snorkel mask, whilst all participants who failed the snorkel mask (n = 6), also failed the FFP3 mask. Within the placebo-exclusion subgroup, all the 8 participants who passed only one mask did so with the snorkel (p = 0.008 for comparison). Of the participants who failed the snorkel mask test 7/14 (50%) had either a beard or glasses on.

### Droplet deposition protection

**Fig 2:**
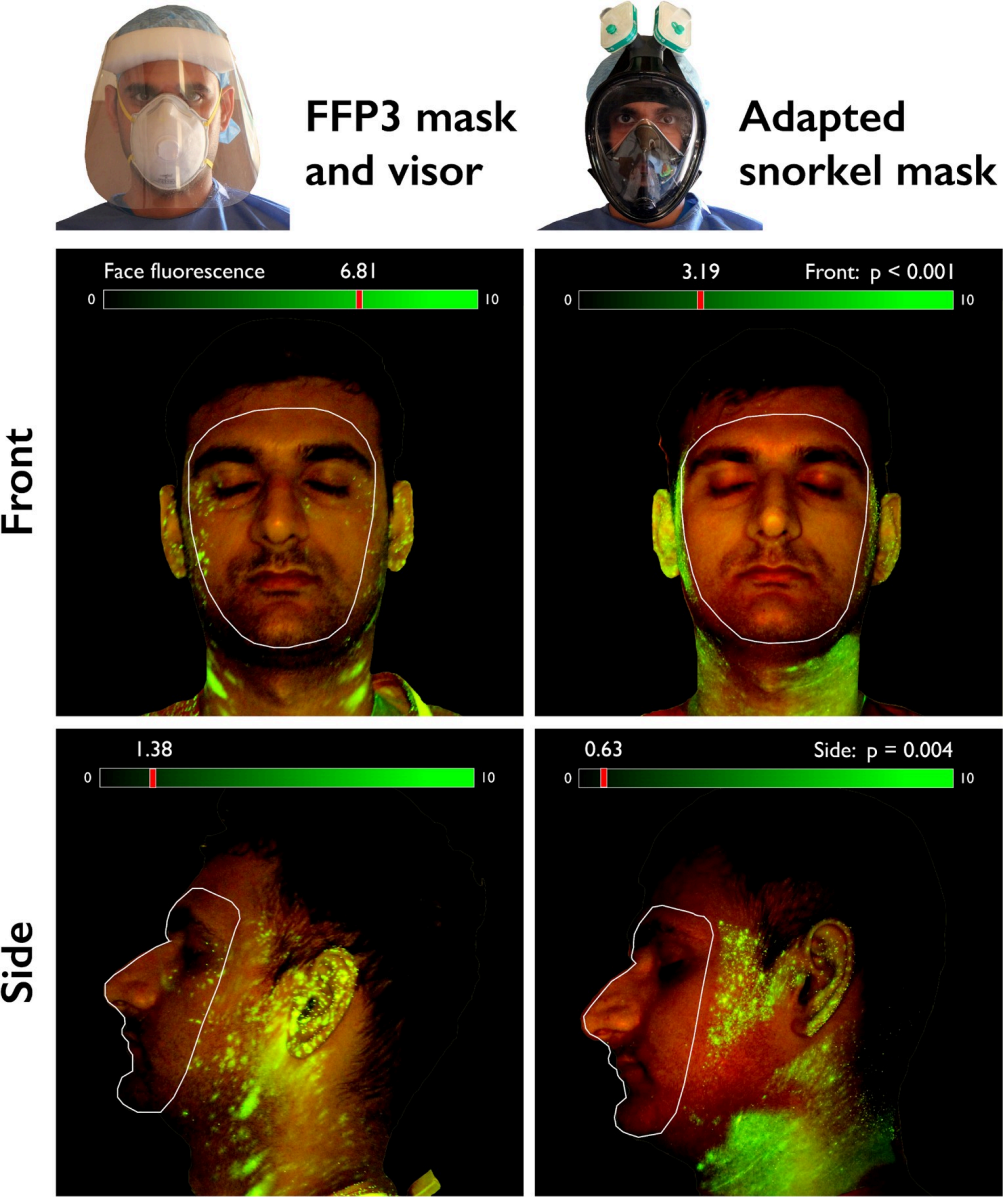
Front and side face UV-light images (with blue filter applied) following spraying of fluorescence oil and doffing of personal protective equipment. The snorkel mask provides better protection against fluorescence droplets, compared to a combination of FFP3 masks and face visor. Fluorescence scale in Corrected Total Fluorescence units (x10^8^) (see also S1 Fig 2 in S1 Appendix for other comparisons).

Following spray of fluorescence solution, the adapted full-face snorkel mask provided better protection against droplet deposition, when compared to a standard guideline-recommended [1, 3] combination of FFP3 mask plus face visor (Fig 2). In each aspect photographed, there was significantly higher face fluorescence following removal of standard PPE compared to the snorkel mask (mean corrected total fluorescence units, front face: 6.81 x 10^8^ vs 3.19 x 10^8^ , p<0.001; left face: 1.38 x 10^8^ vs 6.33 x 10^7^, p = 0.004; right face: 2.70 x 10^8^ vs 5.35 x 10^7^, p = 0.005). The superior protection offered by the snorkel was also observed against the use FFP3 mask and protective glasses (S1 Fig 6 in S1 Appendix).

Other than via fluorescence quantification, it can also be visually appreciated from the acquired images (Fig 2 and S1 Fig 6 in S1 Appendix) that the snorkel mask is particularly effective at preventing deposition of droplets around the participant’s eyes and nose, areas of potential viral entrance.

## Discussion

This study demonstrates that full-faced snorkel masks adapted as particulate filters can be used as a safe alternative to standard PPE combinations of FFP masks and eye protection. Snorkel masks were superior to standard FFP3 masks during standard mask fit testing (Fig 1) and provided better surface protection against sprayed droplets and aerosols when compared to currently recommended PPE combinations (Fig 2 and S1 Fig 6 in S1 Appendix).

The potential benefits of using an adapted snorkel mask as a respirator are several. Firstly, it appears to provide an overall better filtering capacity across all participants tested, when compared to a standard widely used type of FFP3 mask. With rates of failure of FFP masks amongst staff known to be high (32–45% in our study and 26–27% in previous published reports [16, 17]), the snorkel mask appears to suit a wider range of face shapes, providing superior sealing against aerosols reaching the participants face and airways. Secondly, the two main components of our proposed mask are fully washable and reusable: the mask itself is designed to be used under water and can be cleaned with soapy water or chlorhexidine; most 3D printable materials available worldwide can also be re-sterilised; and the particle filters we adapted the snorkel mask to have a long shelf life and can equally be cleaned (3M and HMA filters). Finally, the snorkel mask ($25–40), connector ($2–3) and filter ($5–15) combination is a cost-effective PPE strategy considering their reusability.

Using snorkels as respirators has been previously reported. Work by Greig et al. [18] showed that the wearing of a single-filter adapted snorkel mask was safe for the wearer based on expired levels of CO2 however this result may not be reliable given that quantitative testing identified a possible air leak even during normal breathing. Also, full-face snorkel masks have been adapted to be used as non-invasive ventilation masks for patients, further demonstrating the safety of the system as a particle filter [19]. Visual inspection of the droplet deposition experiment results suggests that the snorkel mask may be effective at preventing deposition of droplets around the participant’s eyes and nose. The clinical relevance of this finding might be that infected droplets deposited closer to the nasal passage could more easily enter the respiratory epithelium, by virtue of having a shorter discharge to be transferred by hand. Studies show that the nose and cheek are among the commonest areas of face-touching [20].

These results are reported with caution, given that they are based only on the mask wearing and removal habits of a single participant, and manual transfer of infected droplets from more peripheral facial areas (e.g. cheeks, ears) is still a common method of virus entrance to the respiratory tract. Importantly, our 3D-printable connectors offer several improvements over previously reported adaptations. Firstly, we reduced ventilatory resistance rates by adapting two filters to each mask. Secondly, we have shown that more than one outflow type can be adapted (elliptical and circular) as well as to more than one filter type (3M filters as well as standard HMA ventilator system filters). Finally, the designs will be available for free download online at https://www.ImperialHackspace.com/COVID-19-Snorkel-Respirator-Project/ with instructions on how to print and adapt the individual pieces (see S1 Appendix for details).

### Other studies supporting our findings

In this study we have sought to assess the adapted snorkel mask in the routine way (qualitative mask fit testing) against the first line alternative (FFP3 masks). Since these experiments were performed other researchers have published studies detailing through testing of adapted snorkel mask respirators.

For example Kroo et al. [21] presented a similar arrangement with a variety of filter configurations attached to a snorkel mask via a 3D printed connector. They demonstrated their masks’ success in qualitative and quantitative fit testing and also reported acceptable work of breathing, valve leakage and carbon dioxide build-up. For the Subea Decathlon mask with an industrial HEPA filter at the level of the mouth chamber the arrangement passed all sections of a half-face respirator mode quantitative test with an overall fit factor (ratio of particles outside the mask to within) of 110.

Germonpre et al. assessed 13 different mask and filter configurations and found overall fit factors ranging between 52 and over 200 with the standard disposable mask respirator based PPE arrangement scoring 62 [22]. The finding is very encouraging in that the masks, filters, connectors and protocols used were comparable to those described here however the mask we present would require its own rigorous quantitative testing prior to CE marking and clinical use.

While Kroo et al. did not assess droplet deposition Chuan et al. [23] used an ophthalmologist’s slit lamp to manually count droplets sprayed onto a mannequin’s face. They showed evidence that physical shields and face masks were effective in reducing droplet deposition. While the PPE assessed was different the method used is related and supports the use of a visually assessed droplet density method which we feel has been refined with the use of quantitative fluorescence and applied to the snorkel mask paradigm. Takes together these studies serve to underline the desire to develop agile strategies to overcome mask scarcity but subject them to the highest level of scrutiny prior to clinical use.

### Limitations

The filtration experiment was performed as part of routine FFP mask fitting testing during the COVID-19 pandemic at our hospital, with no quantitative measurement of filtration rates. This is a valid approach as this is the most commonly used method of mask fit testing in the clinical setting [24]. The method used is dependent on the ability of the human taste pathway to detect the aerosol. This psychophysical method has inherent limitations in that some will not be able to taste the solution ever and others may think they are tasting it when they are not. We have used the placebo test to try and control for this as much as possible.

The droplet deposition experiment cannot tell us exactly how droplets containing virus particles would behave but demonstrates visually and quantitatively that snorkel mask improves face surface cover compared to currently recommended PPE strategies.

The FFP3 control mask used was different for the aerosol filtering test (3M foldable 1873M) and droplet deposition test (Medline NR-EN1 respirator mask). The two experiments demonstrate different, non-overlapping, features of the snorkel mask (protection against aerosols and droplets respectively) and no comparisons between the protection offered by the FFP3 control masks in each experiment are made. In the case of the aerosol filtering test, the best-fitting mask for each participant was unknown prior to the study, so they were tested on the mask that was available in clinical areas at the time. In the case of the droplet deposition test, the participant had previously undergone a qualitative fit-test on both masks, and the Medline mask was found provide the best face-seal. We therefore chose this mask to be worn by them during the experiment, to avoid biasing the results in favour of the snorkel mask by knowingly allowing ingress of fluorescent droplets due a mal-fitting FFP3 mask.

It is possible that the droplet deposition experiment produced very small droplets that were not resolved by the camera sensor and subsequently missed by the image analysis software. We believe that the large size of the camera sensor used mitigated this limitation. Furthermore, since the comparison between masks is only internal to the experiment, both masks would be equally affected by this limitation therefore the comparison of fluorescent particles that *were* resolved by the camera and software is valid.

The FFP3 mask has an expiratory valve and the impact of leakage on the experimental results cannot be quantified here. That said the adapted snorkel mask does contain a valve for expulsion of water. Furthermore, expiratory valves in approved respirator devices must demonstrate minimal leakage in formal testing. The important finding is that the masks both performed well on qualitative testing which, allowing for limitations of the psychophysical method, should capture failure of filtration at any point. If particles are drawn into the mask the participant will taste them.

This study was inspired by a scarcity of PPE during the first wave of the pandemic and accordingly there was a necessary inconsistency in the snorkel masks, disposable masks and filters used in different parts of the study. This only serves to emphasize the importance of the work to identify alternatives however and we believe the generalisability was minimally affected due to the similarity between the items and the rigorous standards that exist. It is important to stress that the qualitative test is a potentially flawed measure of mask fit. It cannot account for all circumstances, work patterns, behaviours and challenges. As well as this, changes in facial hair and glasses are not accounted for and the care and attention that individuals apply when donning, doffing and disposing of PPE. Furthermore, with a changing supply of equipment, a new test is needed for each change in available PPE. The aim of this study was simply to expose the novel adapted snorkel mask to the currently accepted standard of testing on the front line. Prior to clinical use a more rigorous quantitative protocol would be needed.

Finally, we have not tested the tolerability of the snorkel mask by healthcare workers over extended periods. However, we envisage that such adapted device will be used in high risk environments (such as intensive care units or accident and emergency departments) or during aerosol generating procedures, for short periods at a time.

## Conclusions

During COVID-19 and other future global virus outbreaks, full-face snorkel masks adapted as particulate respirators could provide a safe, easily accessible and reusable protection system for healthcare workers. Adapted snorkel masks appear to provide better overall protection against droplets and aerosols than standard, guideline-recommended combinations of FFP3 mask and eye protection.

## Supporting information

S1 Appendix Supplementary appendix with detailed methods. In this supplementary appendix we provide detailed testing protocols and supplementary data from both experiments.
(PDF)

S1 Data Anonymized data from experiments. In this Microsoft Excel document, we present the anonymized data from both experiments. Some of the baseline characteristics (participant role and facial hair/glasses) have been redacted to avoid the identification of participants.
(XLSX)
