# Correction: Reusable snorkel masks adapted as particulate respirators

**DOI:** 10.1371/journal.pone.0257133

**Published:** 2021-09-01

**Authors:** Henry Seligman, Sameer Zaman, David S. Pitcher, Matthew J. Shun-Shin, Freya Hepworth Lloyd, Vitaliy Androshchuk, Sayan Sen, Rasha Al-Lamee, David M. Miller, Harry W. Barnett, Gulam S. Haji, Luke S. Howard, Sukhjinder Nijjer, Jamil Mayet, Darrel P. Francis, Oscar Ces, Nicholas W. F. Linton, Nicholas S. Peters, Ricardo Petraco

The sixth author’s name is spelled incorrectly. The correct name is: Vitaliy Androshchuk. The correct citation is: Seligman H, Zaman S, Pitcher DS, Shun-Shin MJ, Hepworth Lloyd F, Androshchuk V, et al. (2021) Reusable snorkel masks adapted as particulate respirators. PLoS ONE 16(4): e0249201. https://doi.org/10.1371/journal.pone.0249201
