# Supplementary material for: Reusable snorkel masks adapted as particulate respirators
Source: PLoS One. 2021 Apr 5;16(4):e0249201. doi: 10.1371/journal.pone.0249201 (PMC8021169; doi:10.1371/journal.pone.0249201)
Supplement: S1 Appendix — In this supplementary appendix we provide detailed testing protocols and supplementary data from both experiments. (PDF) [file pone.0249201.s001.pdf]

S1. Reusable Snorkel Masks adapted as Particulate Respirators Supplementary Appendix

Table of contents

Detailed Methods

Snorkel masks……………………………………………………………………………… 2
S1 Fig 1…………………………………………………………………………………… 2
Particulate filters…………………………………………………………………………… 2
S1 Fig 2…………………………………………………………………………………… 3
S1 Fig 3…………………………………………………………………………………… 3
Connector design and 3D printing ……………………………………………………… 4
S1 Fig 4…………………………………………………………………………………… 4
Filtering test experimental protocol ……………………………………………………… 5
Materials…………………………………………………………………………………… 5
Preparation………………………………………………………………………………… 5
Sensitivity test……………………………………………………………………………… 5
Fit test……………………………………………………………………………………… 6
S1 Table 1………………………………………………………………………………… 7
Droplet deposition experiment…………………………………………………………… 7
Quantification of fluorescence from still facial images after PPE removal……………… 8
S1 Fig 5…………………………………………………………………………………… 9
Data analysis……………………………………………………………………………… 9
Appendix Results………………………………………………………………………… 10
S1 Fig 6 …………………………………………………………………………………… 14
S1 Table 2………………………………………………………………………………… 11
References………………………………………………………………………………… 12

Detailed methods

Snorkel masks

Two brands of commercially available full-face snorkel masks were used (X99 and Mosfiata both purchased on Amazon). The masks are made of a moulded plastic shell with a silicon sleeve to provide comfort and a water-tight seal. They combine the function of traditional diving mask and snorkel into a single full-face respirator with wide field of view. The masks are held to the face with polyester straps. They have a rigid inflow opening at the top with a detachable snorkel. The two brands had different shaped inflow pipes requiring different shaped connectors to be designed (one round one elliptical). There is a water outflow valve at the front. They have an enclosed breathing chamber around the nose and mouth designed to separate inhaled and exhaled air to prevent retention of carbon dioxide and fogging(1).

S1 Fig 1. The two masks used in the experiment. X99 (L) with round inflow and Mosfiata (R) with elliptical inflow.

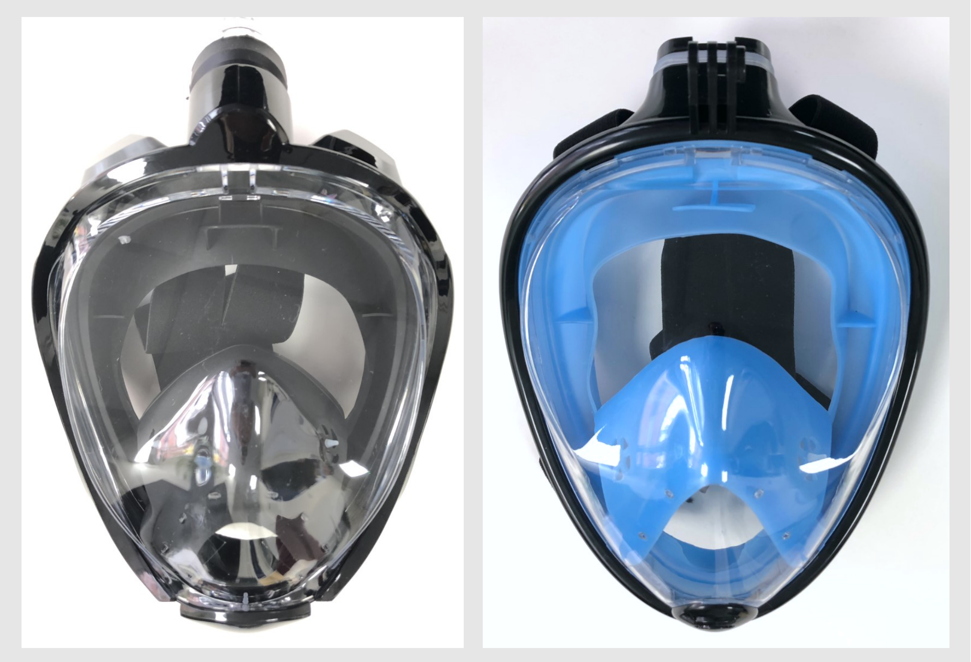

Particulate filters (filtering test experiment)

Two types of filters were used in this is experiment. A 3M 6035 encapsulated filter (3M, Saint Paul, Minesota, USA), designed to be used with half-face or full-face respirator masks. Two are used at any one time attached to a single mask. The filter capsules normally connect via a bayonet-style connector. This filter was chosen because it is globally available and from a widely used and reputable brand. They offer low breathing resistance and have a larger surface area(2).

S1 Fig 2. The 3M 6035 P3 filter

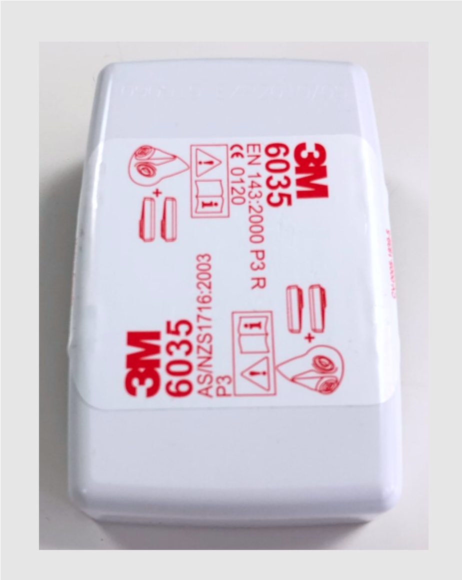

Intersurgical 'Filtatherm' (Intersurgical, Wokingham, UK): these filter attachments are designed to sit in-line within a mechanical ventilator circuit to allow filtration of particulate matter but retention of moisture and heat. They weigh 42g and feature round 22F male and a 22F male/15F female connector. The "patient" side of the filter was attached to the inflow of the snorkel mask using 3D printed adaptors. They have a 99.999% filter efficiency(3). They were chosen because of low price and wide availability in intensive care units globally.

S1 Fig 3. Intersurgical Filtatherm filter

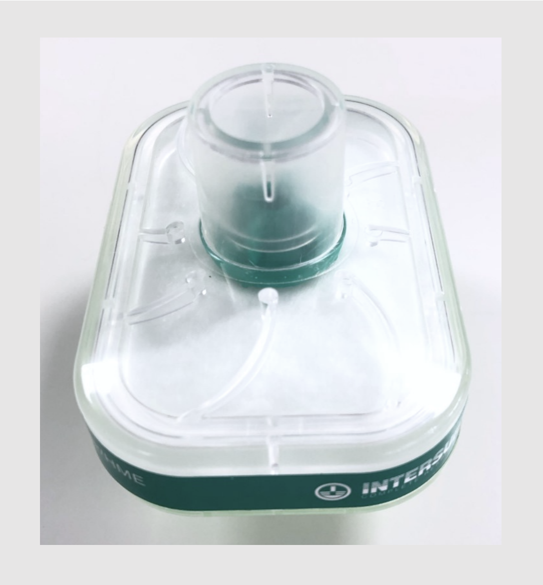

Connector design and 3D printing

Filters were attached to the snorkel masks using bespoke 3D printed connectors. The dimensions were measured manually with high precision rulers and 3D computer aided designs (CADs) were made in Fusion 360 software (Autodesk, Mill valley, California). Connectors were printed using a FormLabs 3 printer (Formlabs, Somerville, Massachusetts) at a resolution of 100 µm layers using Tough 2000 resin (Formlabs, Somerville, Massachusetts). The prints were then washed 2x 10 mins in 100% propyl 2 alcohol before being cured at 70° C for 1 hour. CADs for the connectors and images of the printed pieces are available for free download at: https://www.ImperialHackspace.com/COVID-19-Snorkel-Respirator-Project/

S1 Fig 4. Examples of the 3D printed connectors used in the experiments

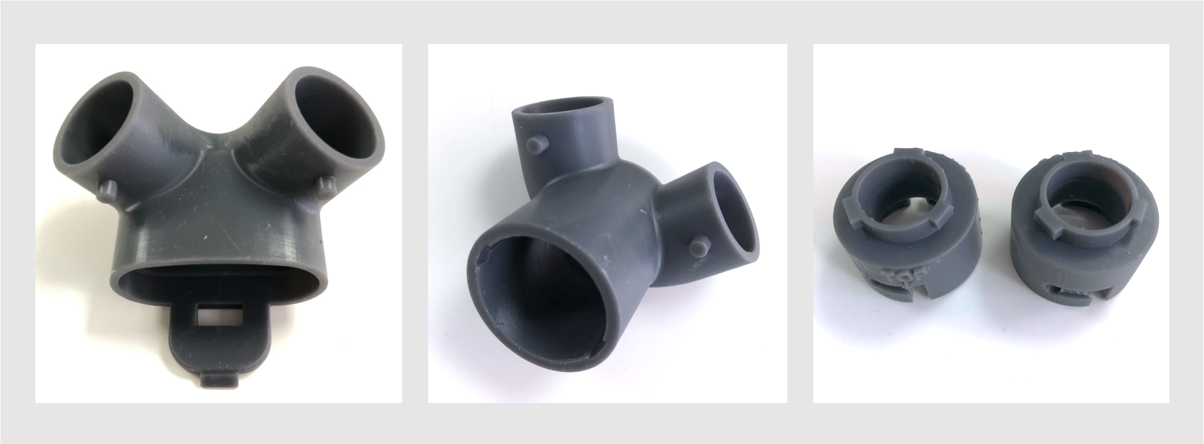

Filtering test experiment mask fit testing protocol

The filtration capacity of the adapted snorkel mask was compared to that of a standard FFP3 mask using a modified version of the standard qualitative respiratory protective equipment (RPE) testing protocol which is routinely used to test the fitting of FFP masks in healthcare workers at our trust (Imperial College NHS Trust)(4). Each was randomly allocated to sequential testing with both snorkel mask and standard FFP3 mask. Following the introduction of aerosolised solutions (bitter denatonium *or* sweet saccharine) and placebo (water) into a hood, participants were asked whether they could taste it, whilst performing a standardised protocol of actions (normal breathing, deep breathing, head movement, speaking and bending). Those reporting to taste the solution at any stage, were considered to have failed the test. Those who tasted the placebo aerosol (false failures) were excluded in a pre-specified analysis.

Materials

A commercially available testing kit (Medline n95 qualitative fit test kit, (Medline, Northfield, Illinois)) should be used. A plastic hood with a transparent window is worn to contain a concentrated cloud of testing solution vapour around the edges of the participants head. Hand pumped nebulisers are used to aerosolise the testing solutions. 3 different solutions are used: Bittrex (denatonium benzoate), saccharine and water. The Bittrex and saccharine are used in two strengths: a weaker sensitivity solution and a stronger testing solution (exact concentrations are not published by the manufacturers).

Preparation

The participant should be seated in a comfortable position, back upright, in a well ventilated room wearing usual work attire. They should have avoided consuming food or drink or smoking for 15 minutes before the test. The test should be explained and participants should be asked for routine information about their demographics, role and previous mask-fit test results. A nebuliser is used which is designed to create fine mists with a 2-5 μm diameter with a 5 ml capacity in accordance with the international standard (ISO 16975-3). 2-3mls of sensitivity solution, testing solution and placebo are poured into separate nebulisers away from the view of the participant.

Sensitivity test

The purpose of this test is to demonstrate the participant can taste the test solution compound and avoid 'false passes' due to insensitivity.

The hood is placed over the head of the participant without a mask and draw-strings and snaps are secured. They should be asked to lean forward slightly to allow the mask to drop forward leaving a gap between their face and its transparent screen (which has a hole to allow the nebulised vapour to enter). The participant is asked to breathe through their mouth throughout.
Vapour is expelled from the nebulizer by fully compressing the rubber bulb. Ten squeezes are given as an initial bolus and the participant is asked if they can taste the testing agent (bitter in the case of Bittrex or sweet in the case of saccharine). They are not informed that placebo is an alternative option. Further squeezes are given up to a maximum of 30. Participants who could not sense the agent by that point are deemed insensitive to it. When the alternative agent is available this is offered to insensitive participants. Those insensitive to the available agent(s) are excluded.

Fit test

Participants don their mask and then the hood. Testing solution is infiltrated into the hood using the appropriate nebuliser according to the level of sensitivity starting with a bolus of multiple squeezes followed by further maintenance squeezes every 30 seconds (see S1 Table 1). In total four combinations of mask and agent are used once sensitivity has been determined:

1. FFP3 and test solution
2. FFP3 and placebo
3. Snorkel mask and test solution
4. Snorkel mask and placebo

The order of the four tests is randomised using free online computerised software (Sealed Envelope, Clerkenwell, London) in a single-blinded fashion.
For each combination the participant performs the following manoeuvres and is asked to state if at any time they taste the testing agent (bitter or sweet).

1. Normal breathing
2. Deep breaths
3. Turn head left and right
4. Look up and down
5. Speaking
6. Bending forwards
7. Normal Breathing

If the participant was able to taste the agent at any time they are deemed to have failed the test for that combination of mask and testing agent. At the end of the four test the participant was asked to remove the hood and mask and the experiment ends.

S1 Table 1. Testing solution doses according to sensitivity

Squeezes required in sensitivity test	Bolus	Maintenance
1-10 (normal)	10	5
11-20 (low sensitivity)	20	10
21-30 (minimal sensitivity)	30	15

Droplet deposition test

Fluorescent droplets observed under ultraviolet (UV) light were used to illustrate spread of hazardous droplet material and cross-contamination during the process of personal protective equipment (PPE) removal.

5 grams of florescent powder (Golden Yellow Fluorescent Powder, Flint Hire & Supply Ltd., London, UK) was suspended in 30ml of low viscosity oil (Johnson's baby oil, Johnson & Johnson GmbH, Neuss, Germany). An oil-based medium was used to avoid evaporation and subsequent loss of vibrancy. The suspension was placed in a handheld trigger spray bottle. The spray bottle's nozzle was adjusted to produce a fine mist of droplet spray, visibly analogous to fine droplet sprays produced by coughing or sneezing.

The participant, dressed in surgical scrubs, surgical tie-back hat and full sleeved surgical gown donned PPE in accordance with Public Health England Guidelines(5). The following combinations of facial PPE were used. FFP3 mask in this experiment refers to Medline NR-EN1 respirator mask, (Medline, Northfield, Illinois):

(a) FFP3 mask and protective visor
(b) FFP3 mask and protective glasses
(c) FFP3 mask, protective visor and protective glasses
(d) Modified snorkel mask with two 3M mask filters

The participant was sprayed by a clinical researcher at a distance of 50cm, with two pumps of the handheld spray, in each of four positions (the participant rotated on the spot rather than the sprayer moving):

(a) Front of head
(b) Right side of head
(c) Back of head
(d) Left side of head

The participant was photographed using a Canon EOS 700D DSLR camera and Canon EF-S 18-55mm lens (Canon Inc., Tokyo, Japan). For the pictures under UV light, a battery-powered 68 LED bulb, 395nm handheld UV flashlight was used (Lighting Ever Ltd., Birmingham, UK). PPE was removed in accordance with Public Health England guidance(5). For each set of PPE above, the following photographs of the participant's head were taken.

In daylight

(a) PPE on, before spray (front)

Under UV light

(a) PPE on, before spray (front)
(b) PPE on, after spray (front, left, right)
(c) PPE removed, after spray (front, left, right)

For standardisation, the photographs were cropped to 1000 x 1000-pixel size and a plain black background was placed for UV photographs using photo editing software (GNU Image Manipulation Program 2.10.14, The GIMP development team, California, USA; Adobe Illustrator CS 6, Adobe Inc., Delaware, USA).

Quantification of fluorescence from still facial images after PPE removal

The degree of fluorescence in still facial images after doffing PPE was analysed using biological-image analysis software (ImageJ on the Fiji distribution(6)). Each colour image was split into its constituent RGB stack. The G (green) channel was selected for analysis because this isolated the part of the colour spectrum relevant to the colour of fluorescent powder used in the experiments. A pre-determined region of interest (ROI) was selected for analysis. This was 1cm above the eyebrows superiorly, 1cm lateral to the eyebrows laterally and the tip of the chin inferiorly. Following the protocol adapted by Hammond(7), a background reading was collected from a 100 x 100 pixel area devoid of fluorescence near to the ROI. Corrected total fluorescence (CTF, arbitrary units) was derived using the equation below: [2]

CTF = Integrated density – (Area of selected region of interest X mean fluorescence of background readings)

The region of interest and background readings were drawn on three separate occasions for each picture and their means and standard deviations were calculated. For each aspect (front, left, right), the difference between the mean CTF in photographs after the removal of the adapted snorkel mask and FFP3 mask with visor were compared using an unpaired t-test.

The following quantity definitions were used:

Area of ROI: The size, in pixels, of the region of interest that has been manually drawn.

Integrated density: The product of area and mean intensity (where mean intensity is the mean grey value inside the region of interest).

Fluorescence of background readings: Calculated to calibrate the final result against the background natural fluorescence of the environment by drawing three regions of interest from the image background (away from the participant’s face) and counting

the mean grey value inside those three background ROIs. This is then multiplied by the ROI area and subtracted from the integrated density.

S1 Fig 5. Example regions of interest (ROI) for quantification of fluorescence

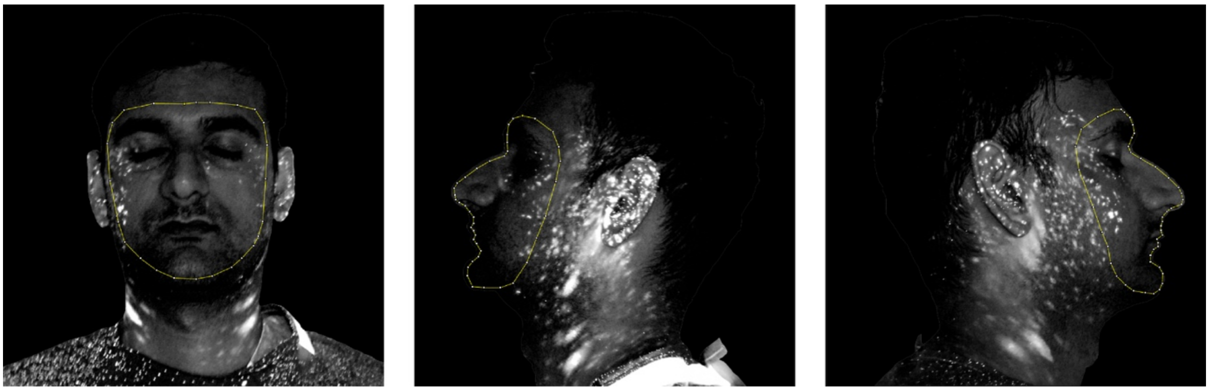

Data analysis

Results of filtering test pass rates are expressed as proportions and percentages. The McNemar test was used to assess the differences in proportions of pass or fail rates between the two masks. Fluorescence data was presented as mean corrected total fluorescence units. An independent samples t-test was used for comparison.

Appendix Results

Full results of the droplet deposition experiment are given in S1 Fig 6 and S1 Table 2.

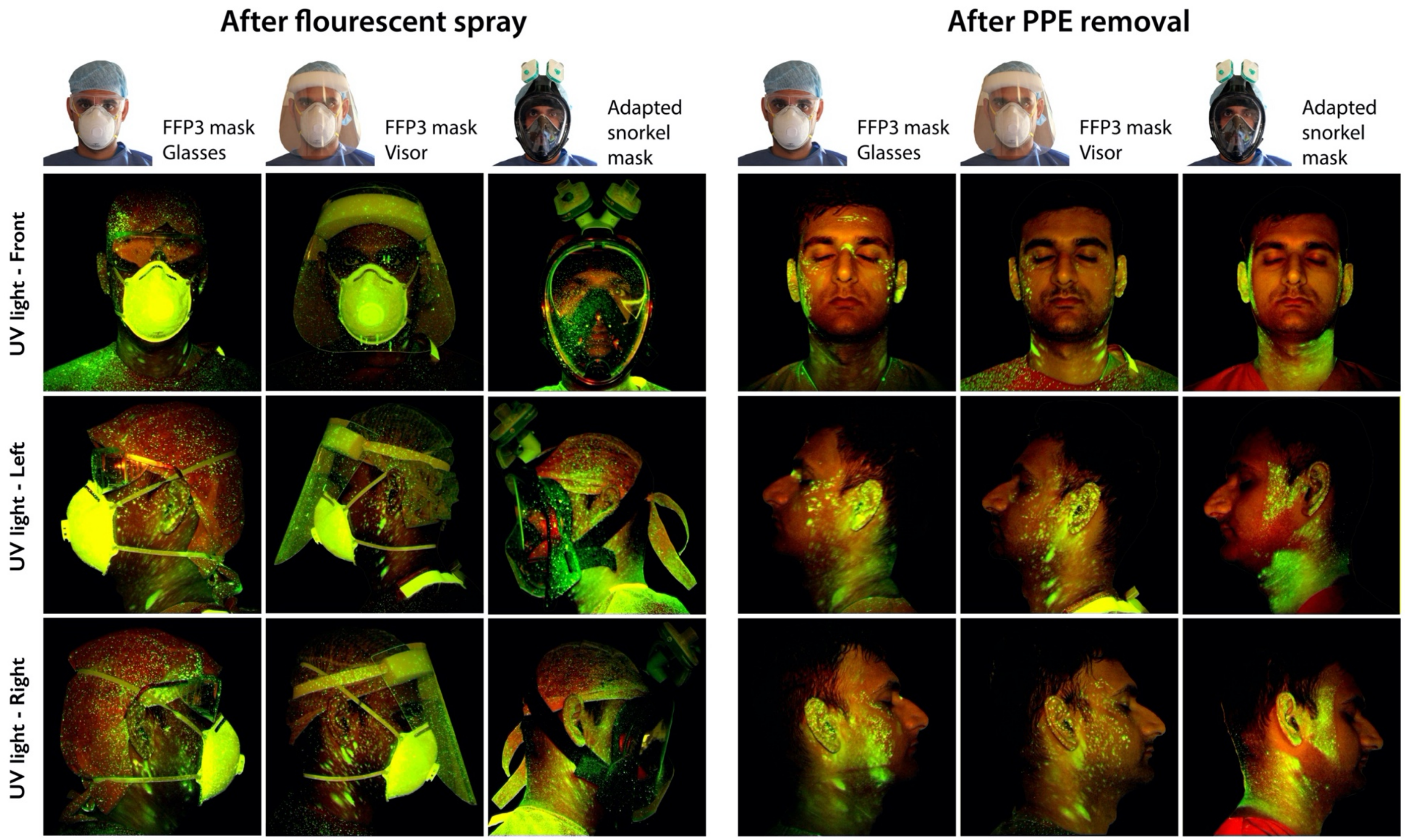

S1 Fig 6. The adapted snorkel mask compared to two different PPE arrangements (FFP3 mask with glasses and FFP3 mask with visor). It can be seen that there are more droplets around the nose and mouth of the participant in both the routine PPE arrangements which are not seen in the case of the adapted snorkel mask.

S1 Table 2.

View	FFP3 mask and glasses (A)	FFP3 mask and visor (B)	Adapted snorkel mask (C)	p value A vs B	p value A vs C	p value B vs C
Front CTF (units)	6.81×10^8	7.63×10^8	3.19×10^8	0.037	<0.001	<0.001
Left CTF (units)	1.38×10^8	2.04×10^8	0.63×10^8	0.008	<0.001	0.004
Right CTF (units)	2.70×10^8	2.99×10^8	0.54×10^8	0.573	0.001	0.005

Abbreviations, FFP: Fixed face piece, CTF: Corrected total fluorescence

References

1. X99. X99 Snorkel Mask Foldable 180 Panoramic View Free Breathing Full Face Snorkeling Mask with Detachable Camera Mount, Dry Top Set Anti-fog Anti-leak for Adults & Kids (Blue black frame-kids, XS) [Internet]. www.Amazon.co.uk. 2020 [cited 2020 Jun 15]. Available from: https://www.amazon.co.uk/X99-Panoramic-Breathing-Snorkeling-Detachable/dp/B0841YC3SL
2. 3M. 3M technological Bulletin [Internet]. www.3m.co.uk. 2009 [cited 2020 Jun 11]. Available from: https://www.3m.co.uk/intl/uk/ohes/segments/healthcare/(9666a)OH_ReusableTechBulletin_lft.pdf
3. Intersurgical. Filta-Therm Product Characteristics [Internet]. www.intersurgical.com. 2020 [cited 2020 Jun 2]. Available from: https://www.intersurgical.com/products/airway-management/filtatherm-range-high-efficiency#1941001
4. FOD Central Specialist Division. Health and Safety Executive Operational Circular 282/28. Health And Safety Executive; 2012 Apr p. 44.
5. Public Health England. New personal protective equipment (PPE) guidance for NHS teams [Internet]. www.gov.uk. 2020 [cited 2020 Apr 27]. Available from: https://www.gov.uk/government/news/new-personal-protective-equipment-ppe-guidance-for-nhs-teams
6. Schindelin J, Arganda-Carreras I, Frise E, Kaynig V, Longair M, Pietzsch T, et al. Fiji: an open-source platform for biological-image analysis. Nat Methods. 2012 Jun 28;9(7):676–82.
7. Hammond L. Measuring cell fluorescence using ImageJ [Internet]. https://theolb.readthedocs.io/. 2014 [cited 2020 Jun 15]. Available from: https://theolb.readthedocs.io/en/latest/imaging/measuring-cell-fluorescence-using-imagej.html
